# Aroma Potential of a New Maltose-Negative Yeast Isolate

**DOI:** 10.3390/foods14193357

**Published:** 2025-09-28

**Authors:** Selin Yabacı Karaoğlan, Rudolf Jung, Lukáš Jelínek, Marcel Karabín, Tomáš Kinčl, Pavel Dostálek

**Affiliations:** 1 Department of Food Engineering, Faculty of Engineering, Adana Alparslan Türkeş Science and Technology University, 01250 Adana, Türkiye; syabaci@atu.edu.tr; 2 Department of Biotechnology, Faculty of Food and Biochemical Technology, University of Chemistry and Technology, Prague, Technická 5, CZ-16628 Prague, Czech Republic; jungu@vscht.cz (R.J.); lukas.jelinek@vscht.cz (L.J.); marcel.karabin@vscht.cz (M.K.); tomas.kincl@vscht.cz (T.K.)

**Keywords:** non-alcoholic beer, aroma, volatiles, carbonyl compounds, *Saccharomycodes ludwigii*, SPME-GC/MS

## Abstract

Non-alcoholic beer is increasingly popular worldwide but still faces flavor challenges compared to regular beer. These flavor-related challenges include pronounced ‘wort-like’ notes, excessive sweetness, and a lack of desirable aroma complexity. The industry is trying to improve the taste of non-alcoholic beer by trying new techniques and yeasts. A newly isolated maltose-negative brewer’s yeast (M-I) from an industrial-scale brewery collection has attracted attention due to its reduced wort-like flavor. This study aims to characterize the volatile profile of a newly isolated maltose-negative brewer’s yeast (M-I) in comparison with the well-known *Saccharomycodes ludwigii*. The novelty of this work lies in evaluating the aroma potential of a maltose-negative isolate newly applied in industrial brewing and its contribution to improving the flavor quality of non-alcoholic beer. An SPME-GC/MS system was used to analyze aroma compounds. According to volatile compound analysis, the M-I sample has higher amounts of esters and higher alcohol composition than the *S. ludwigii* beer sample. Also, it has lower amounts of Strecker aldehydes, which can give a worty off-flavor. Sensory analysis revealed that, interestingly, the control *S. ludwigii* sample was rated as having stronger ester notes, along with more pronounced sour and bitter characteristics, whereas the M-I sample was perceived as having a more balanced flavor, leading to a more favorable rating by the panelists.

## 1. Introduction

Non-alcoholic beer is gaining more and more attention due to increased awareness of health and safety and government policies regarding alcohol. Non-alcoholic beer is defined as beer with less than 0.5% alcohol by volume (ABV) in most European countries and the USA. However, in some countries, beer may be required to contain less than 0.05% alcohol to be alcohol-free [1,2]. The quality expectation of consumers for beer is shaped by many factors, such as foam, color, aroma, mouthfeel, and aftertaste [3]. Consumers expect non-alcoholic beers to have a flavor profile similar to that of regular beers produced by alcoholic fermentation. Aroma, which is one of the most critical factors in the perception of beer flavor, is determined by hundreds of compounds [4]. A large part of beer aroma and taste compounds are formed during yeast fermentation, and therefore, the type of yeast used and its metabolism are highly correlated with the profile of compounds [5]. Alcohol is a crucial flavor component in beer and is naturally formed during fermentation. However, non-alcoholic beers still face some challenges regarding flavor, which vary depending on the production method. These problems include “wort-like” flavors, sweet taste, or lack of aroma [6,7]. These defects may result from a lack of alcohol and the lack of aldehyde-reducing effect of alcohol fermentation, as well as production techniques.

The most preferred commercial non-alcoholic beer production methods are limited fermentation using low-extract wort or dealcoholization of original beer by vacuum distillation [8]. In limited fermentation, yeast performs partial fermentation. Production can be performed with the same equipment in a standard brewery. After a point, yeasts are removed from the media, and fermentation stops. Because it is not fully fermented, the final beer is poor in aromatic compounds and has a worty flavor [9]. Generally, beer produced with this method is corrected with the addition of some aroma compounds like isoamyl acetate (banana) to overcome the worty off-flavor [10]. In the distillation method, the alcohol produced by fermentation is separated from the regular beer. It requires significant investments in specialized equipment to separate the alcohol. After optimizing the separation process, the sensory quality of the non-alcoholic beer produced using distillation methods is generally acceptable. However, aroma losses and baked-like odor differences occur using thermal systems to aid in the separation [1,2]. Nowadays, technologies using maltose-negative yeasts can also be applied without extra investment costs in a regular brewery. These special yeasts cannot ferment maltose, the primary sugar of wort. This method is seen as a more palatable and sustainable method [11]. Brewers and researchers are working to find new commercially applicable maltose-negative yeasts from alternative sources. *Saccharomycodes ludwigii* is the most commonly studied yeast in the context of low- and non-alcoholic beer, and it has seen notable industrial and scholarly attention. *Saccharomycodes ludwigii* has been patented [12,13], used in the industry [14], the subject of most studies [15,16,17,18,19] and accepted as a reference for many low- and non-alcoholic beer studies today [11,18,19,20].

Recently, a new yeast used in the production of commercial non-alcoholic beer has attracted attention with its performance. Thus, this work aims to reveal the volatile structure of the new maltose-negative brewer’s yeast isolate (M-I) and compare it with a known *S. ludwigii* strain.

## 2. Materials and Methods

### 2.1. Yeast Strains and Fermentation Trials

Sample beers (4 °C) were produced in an industrial-scale brewery using a maltose-negative yeast strain (M-I) obtained from the brewery’s yeast collection, together with the well-known *S. ludwigii*, under the same wort and production conditions. *S. ludwigii* (NCYC 730) was purchased from NCYC—National Collection of Yeast Cultures (Quadram Institute Bioscience, Norwich Research Park, Norwich, UK). This strain, *S. ludwigii*, was originally isolated from grape must in Germany. Maltose-negative strain (M-I) is a *Saccharomyces* yeast strain also historically isolated from grape must in the Czech Republic and is now collected in commercial private breweries’ yeast collections. Wort pH was 5.0 ± 0.2, and the original extract was 5.4 ± 0.3% *w*/*w* Bitterness was determined to be 30.0 (±2) BU. Pilsen-type barley malt was used for mashing, and type 90 pellets from the Sladek hop variety were used for bittering. Fermentation was conducted at 30 °C for 24 h and 48 h at 10 °C. Maturation was performed at 2 °C for one week. The final beer was saturated with CO_2_ to 5 g/L.

### 2.2. Analytical Determinations

Beers were degassed by an ultrasound bath (10 °C, 10 min). Then, the original extract, apparent extract, pH, alcohol content, and color of beers were measured using a DMA 4500 M Beer Alcolyser (Anton Paar, Graz, Austria) according to the principle of ASBC Methods for beer analysis [21]. All analyses were carried out three times, and the average values were presented with absolute deviations.

For SPME-GC/MS, 50 mL of the beer was degassed and centrifuged. From these samples for volatile compounds analysis, 10 mL were then added to a 20 mL dark glass vial containing 2 g NaCl, 100 µL of an internal standard solution (ethyl heptanoate 11.68 µg/L (≥99%, Sigma-Aldrich, St. Louis, MO, USA), and 3-octanol 21.83 µg/L (≥99%, Sigma-Aldrich, USA) in 70% (*v*/*v*) ethanol/water solution) and sealed with a PTFE-silicone septum (Supelco; Bellefonte, PA, USA), then stirred for 1 min on a vortex to dissolve the salt. Volatiles were analyzed by Agilent GC 6890N—Agilent Technologies, Santa Clara, CA, USA, equipped with a single quadrupole mass spectrometer detector (Agilent 5975B, Inert MSD—Agilent Technologies, USA). The gas chromatograph was coupled to a headspace solid-phase microextraction (HS-SPME) autosampler (COMBI PAL CTC Analytics, Unterägeri, Switzerland) using an 85 µm Carboxen^®^/Polydimethylsiloxane (CAR/PDMS) SPME fiber (Supelco, USA). The isolation was performed for 40 min at 50 °C in an agitator. The extraction conditions were set according to a method published by Nešpor et al. [8]. Separation was carried out on a DB-624 column (30 m × 0.25 mm × 1.4 μm; Agilent Technologies, USA). Carbonyl compounds were analyzed separately due to their low perception thresholds and high sensory relevance as contributors to off-flavors in non-alcoholic beers. In addition, derivatization with o-(2,3,4,5,6-pentafluorobenzyl) hydroxylamine hydrochloride (PFBOA) is required for accurate detection, which necessitates a distinct analytical procedure. For the analysis of carbonyl compounds, 10 mL of a degassed and centrifuged sample, 2 g NaCl (Penta, Praha, Czech Republic), and 50 µL internal standard (56.2 mg/L 3-fluorobenzaldehyde) (Sigma-Aldrich, USA) were added to a 20 mL dark glass vial. The derivatization agent for each analysis was prepared in the same way as the samples, containing 2 g of NaCl, 10 mL of demineralized water (Mili-G Millipore, Millipore, Burlington, MA, USA), and 200 µL of a 5978 mg/L solution of PFBOA (Fluka, Neu-Ulm, Germany; ≥99% purity). All vials were sealed with a PTFE-silicone septa (Supelco, USA) and stirred for 1 min to dissolve the NaCl and to homogenize the sample and derivatization reagent solution. All parameters and the procedure were under the method of [22]. The carbonyl compounds were isolated by Agilent GC 8890N (Agilent Technologies, USA) equipped with a single-quadrupole mass spectrometer detector (Agilent 5977B GC/MSD—Agilent Technologies, USA). The gas chromatograph was coupled to a headspace solid-phase microextraction (HS-SPME) autosampler (PAL RSI 85, CTC Analytics, Zwingen, Switzerland). For GC separation, a DB-5MS column 60 m × 0.25 mm × 1 µm (Agilent Technologies, USA) was used.

The compounds were identified by comparing retention times with standards and their mass spectra with standard mass spectra as listed in the NIST MS spectral database (National Institute of Standards and Technology, Gaithersburg, MD, USA). Quantification was performed from normalized peak areas using an internal standard, by three-point external calibration via the addition of different amounts of external standards: ethyl acetate (99.7%), propyl acetate (≥98%), isobutyl acetate (≥99%, Sigma-Aldrich, USA), isoamyl acetate (98%, Honeywell, Offenbach, Germany), 2-phenylethyl acetate (≥99%), ethyl butyrate (≥98%), ethyl hexanoate (≥99%), ethyl octanoate (≥98%), ethyl decanoate (≥99%) (Fluka, Germany), 2-methyl-1-propanol (≥99%), 3-methyl-1-butanol (≥98.5%), 2-methyl-1-butanol (≥98%), 2-phenylethanol, linalool (≥97%); (Fluka, Germany), 2-methylpropanal (≥99%), 2-methylbutanal (≥95%), 3-methylbutanal (≥97%), (2E)-nonenal (≥98%, Sigma-Aldrich, USA), benzaldehyde (≥98%, Alfa Aesar, Haverhill, MA, USA), heptanal (≥97%), octanal (98%, Merck, Darmstadt, Germany), Diacetyl (97%), Pentadion (2,3 Pentandion), hexanoic (caproic) acid (≥98%), octanoic (caprylic) acid (≥99.5%), (Sigma Aldrich, USA), decanoic (capric) acid (≥99%) (Alfa Aesar, USA).

### 2.3. Sensory Analyses

The triangular test was used to demonstrate a sensorial difference between the M-I and the control sample, *S. ludwigii.* The test followed the American Society of Brewing Chemists ASBC—Methods of Analysis, Sensory Analysis-7 [23].

The test was conducted with 18 trained panelists with prior experience in beer sensory evaluation, who were selected based on their ability to discriminate basic tastes and aromas in pre-tests. Samples were presented randomly to prevent bias. The samples were evaluated under controlled sensory conditions, including neutral lighting, temperature, and noise levels. Each panelist was instructed to cleanse their palate with water between samples to avoid carryover effects.

A confidence level of 95% (*p* < 0.05) was applied to evaluate the test’s conclusiveness.

In addition to the triangular test, a hedonic scale was used to evaluate each sample’s overall preference. The panelists rated the samples on a 9-point scale, where 1 represented “excellent” and 9 represented “horrible.”

A sensory profile test assessed the detailed sensory characteristics of the M-I and control sample (*S. ludwigii*) [23]. Descriptive Analysis (International Method). American Society of Brewing Chemists. The profile test was conducted with 18 trained panelists, who evaluated each sample based on critical sensory attributes, including sweetness, sourness, bitterness, body, estery aroma, hoppiness, maltiness, astringency, and worty characteristics. Each attribute was scored on a scale from 0 to 6, where 0 indicated the absence of the attribute, and 6 represented the highest intensity.

The panelists conducted the evaluation in individual booths under standardized sensory conditions, with controlled lighting and temperature. Each panelist was instructed to cleanse their palate with water between sample tastings to minimize carryover effects. The average score for each attribute was calculated across all panelists, resulting in a sensory profile for both the M-I and control samples.

### 2.4. Statistical Analysis

Statistica 14 software (TIBCO software, Santa Clara, CA, USA) was used to perform the statistical analysis of the chromatographic and sensory data.

## 3. Results and Discussion

### 3.1. Analytical Determination Results

The primary physiochemical parameters of non-alcoholic beers obtained are presented in Table 1. The difference in alcohol content between M-I (0.42% vol.) and *S. ludwigii* (0.35% vol.) is likely due to variations in sugar utilization. Although both fermentations started with a similar original gravity, M-I produced more alcohol because it fermented a greater portion of the available sugars, as indicated by its lower apparent extract (4.69% vs. 4.84%). Additionally, the lower final density of M-I (1.01592 vs. 1.01679 g/cm^3^) suggests a higher fermentation efficiency; being maltose-negative might have left more residual sugars unfermented or directed them toward byproducts like glycerol rather than ethanol. Further analysis of sugar metabolism and byproduct formation could provide deeper insights into these differences. Importantly, even small variations in alcohol content, residual sugars, and glycerol can influence flavor perception, as they are known to affect sweetness, body, and mouthfeel in non-alcoholic beers [24]. Such compositional differences may therefore contribute to perceptible variations in flavor balance between M-I and *S. ludwigii* beers, beyond their role in fermentation efficiency.

Non-alcoholic beers were analyzed by gas chromatography to evaluate how the M-I could affect the final aroma composition compared to the well-known yeast *S. ludwigii*. Aroma analysis results are shown in Table 2. Each compound’s flavor and perception threshold values are also in the same table. It should be noted that these threshold values can provide information about that compound’s impact on flavor. However, when the total beer flavor is considered, it should not be forgotten that apart from the individual effects of these compounds, synergistic effects should also be taken into account [25].

**Figure 1:**
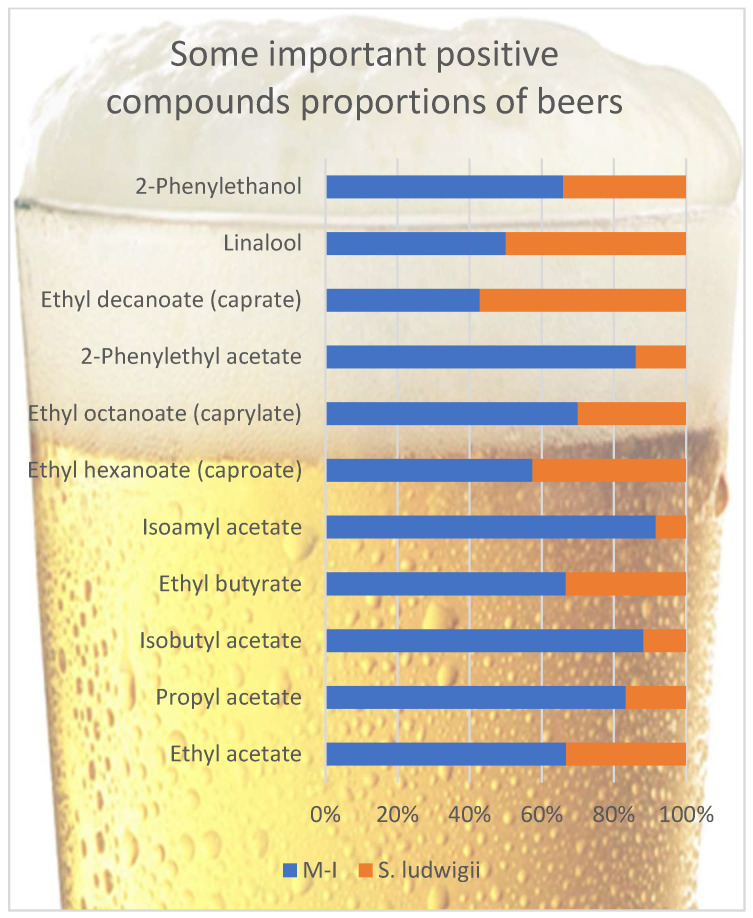
Comparison of some positive volatile compounds in beer samples.

With their low sensory thresholds, esters make a remarkable aroma contribution to beer flavor. A big part of esters is flavor-active compounds that confer a fresh, fruity-flowery aroma [25,32,40]. Esters can be divided into two groups. The first group is acetate esters, like isoamyl acetate (banana), ethyl acetate (solvent-like), phenyl ethyl acetate (roses, honey aroma), and C6–C10 medium-chain fatty acid ethyl esters, including ethyl hexanoate (anise seed, apple-like aroma), ethyl octanoate (sour apple aroma), and ethyl decanoate [40]. Esters are formed during fermentation through the condensation of ethanol or higher alcohols with carboxylic acids or their derivatives [9], and their production largely depends on the yeast strain involved in the fermentation [41]. Other parameters that affect the synthesis of esters are pressure (negatively correlated), temperature (positively correlated), pH (lower pH values may increase ester levels in beer), pitching rate of yeast (positively correlated), wort nutritional concentration- the type of barley (but an increase in sucrose or starch amounts in wort can lead to a loss in the formation of esters) dissolved oxygen concentration in wort (for proper yeast growth) [8]. Figure 1 compares some positive volatile compounds in non-alcoholic beer samples. A big part of these volatiles are esters. The amount of these positive volatile compounds is significant in non-alcoholic beers. Besides their favorable sensory properties, these compounds could also reduce the perception of off-flavored volatiles [42,43]. This is why flavored, semi-dark, non-alcoholic beers are so popular these days.

At first glance, when the ester ratios of the two beers are examined, it is immediately apparent that most of the esters of the M-I samples are in very high amounts compared to the control *S. ludwigii* samples. Exceptionally, ethyl decanoate and ethyl hexanoate were found in amounts close to those in the M-I sample compared to the *S. ludwigii* sample. Ethyl acetate is typically present in the highest concentration of all esters in beers [25]. Ethyl acetate, ethyl butyrate, and ethyl octanoate samples were found in approximately twice the amounts in M-I samples, while other compounds were found more than twice. These compounds generally have desirable fruity-floral flavors and are essential in the beer flavor with apple, citrus, black currant, banana, and rose notes [44,45,46]. It can be seen that most of the esters were found under the threshold value, but the presence of different esters, even under the olfactory threshold, can have a synergistic effect together and, therefore, affect the final beer flavor [32]. Xu et al. [29] examined the contribution of some esters to the flavor of beer in their study, in which the amount of ethyl octanoate was up to 0.41 mg/L (M-I 0.14-*S. ludwigii* 0.06 mg/L), and for ethyl butyrate up to 0.16 mg/L (M-I 0.10-*S. ludwigii* 0.05 mg/L) and phenylethyl acetate up to 1.64 mg/L (M-I 0.31-*S. ludwigii* 0.05 mg/L) have reported that they contribute positively to beer flavor. It is seen that the values in this study do not exceed the maximum values specified as excessive. For isobutyl acetate, 0.96 mg/L was mentioned as the most liked amount, but no limits were declared [29]. The isoamyl acetate ratio, which gives banana notes, was not detected at undesirable amounts. Kinčl et al. [47] showed that about 0.77 mg/L was a more desirable value. The solvent character has been reported to come to the fore at higher values. Ethyl hexanoate amounts in both beers are higher than their threshold value, contributing to the sour apple and aniseed character, which could be sensed in both beers. Recent brewing studies have shown that non-conventional maltose-negative yeasts can produce an intensified ester profile. For example, Johansson et al. [18] found that while *S. ludwigii* beers exhibited only mild fruity notes (apple/pear hints from low 3-methylbutyl acetate), beers brewed with alternative isolates had significantly greater acetate ester concentrations, with certain strains producing 2-phenylethyl acetate above its flavor threshold, imparting strong rose and honey aromas. This suggests that M-I possesses a more active Ehrlich pathway and higher alcohol acetyltransferase activity, leading to elevated formation of isoamyl alcohol (3-methylbutanol) and its acetate (banana-like isoamyl acetate) as well as ethyl esters (e.g., ethyl hexanoate with apple-like notes) relative to *S. ludwigii*. Indeed, in pilot fermentations, a novel maltose-negative yeast yielded a beer with a much more pronounced fruity aroma intensity than an *S. ludwigii*–fermented beer under the same conditions [18].

Higher alcohols are significant contributors to beer aroma. Their concentration is related to the uptake efficiency of the corresponding amino acid and the sugar utilization rate, which are highly strain-specific parameters [15]. Other parameters that affect the synthesis of higher alcohols are pH (lower pH values may reduce levels of higher alcohols), pressure (positively correlated), temperature (positively correlated), pitching rate of yeast (positively correlated), wort nutritional concentration, the type of barley (an increase in sucrose or starch in wort can lead to a loss in the formation of esters and higher alcohols), and dissolved oxygen concentration in wort (for proper yeast growth) [8]. All higher alcohols were found in higher amounts in M-I beer than *S. ludwigii* beer. Among them, inducing an ‘alcoholic and fruity’ flavor, 3-methyl-1-butanol and 2-methyl-1-butanol are essential for beer aroma. With the imparting of alcoholic flavor, 2-methyl-1-propanol may cause a “rough” flavor and harshness in beer. Additionally, 2-phenylethanol contributes positively to the beer aroma with its ‘rose-sweet’ flavor [25]. The higher concentrations of fusel alcohols (e.g., 2-methyl-1-propanol, 3-methyl-1-butanol, and 2-phenylethanol) observed in the M-I sample compared to *S. ludwigii* can be explained by its enhanced amino acid catabolism and fermentation activity. Yeasts produce higher alcohols (fusel alcohols) primarily via the Ehrlich pathway, where amino acids are transaminated to α-keto acids, then decarboxylated to aldehydes and reduced to alcohols [48]. A strain that takes up amino acids more efficiently or in greater excess will channel more of them into this catabolic pathway rather than using them solely for biomass. The M-I strain likely possesses a robust Ehrlich pathway: amino acids such as leucine, isoleucine, valine, and phenylalanine can be absorbed and converted into their corresponding higher alcohols (e.g., isoamyl alcohol from leucine, active amyl alcohol from isoleucine, isobutanol from valine, and 2-phenylethanol from phenylalanine) at higher rates. In contrast, *S. ludwigii* may assimilate these amino acids less into the Ehrlich route—for example, by incorporating a greater fraction into cellular proteins or simply not uptaking them as aggressively—resulting in lower fusel alcohol formation. Notably, once the amino acid-derived α-keto acids are formed in yeast, they cannot be diverted into central carbon metabolism and are instead converted into fusel alcohols or acids [49]. The M-I strain’s metabolism appears biased towards reducing these intermediates to alcohols, which would lead to an accumulation of higher alcohols in its beer.

Strain-specific differences in fermentation kinetics and metabolic regulation further explain why M-I produces more higher alcohols. The M-I yeast ferments the wort sugars more completely and rapidly than *S. ludwigii*, evidenced by its higher ethanol yield and lower residual extract in the beer. Faster and more extensive sugar utilization correlates with elevated fusel alcohol production [50]. This is partly because vigorous fermentation and growth create conditions (e.g., high metabolic flux and early depletion of preferred nitrogen sources) that favor the Ehrlich pathway. Moreover, genetic and regulatory factors innate to the M-I strain may enhance fusel alcohol biosynthesis. Variations in genes governing amino acid transport and metabolism can significantly impact higher alcohol output [51]. For example, high expression or activity of amino acid permeases (for uptake) and key Ehrlich pathway enzymes (e.g., amino acid transaminases and decarboxylases) would promote greater fusel production. In *S. cerevisiae*, the general amino acid permease gene AGP1 and nitrogen metabolism regulators (e.g., GAT1, which controls nitrogen catabolite regulation) are known to influence fusel alcohol levels [51]. It is plausible that the M-I strain is less constrained by nitrogen catabolite repression or has mutations that deregulate amino acid degradation pathways, thereby funneling more amino acid flux toward fusel alcohol formation. In summary, the M-I strain’s metabolic profile—characterized by efficient amino acid uptake, vigorous sugar fermentation, and a potentiated Ehrlich pathway—provides a biochemical rationale for its higher production of fusel alcohols compared to *S. ludwigii* beer. Such enhanced production of esters and higher alcohols by M-I is likely due to species-specific metabolic traits—for instance, some wild yeast isolates natively overproduce fruity acetate esters to attract insects in nature—and it aligns with the trend that many non-*Saccharomyces* strains can generate a richer bouquet of desirable volatile compounds than *S. ludwigii* [52].

**Figure 2:**
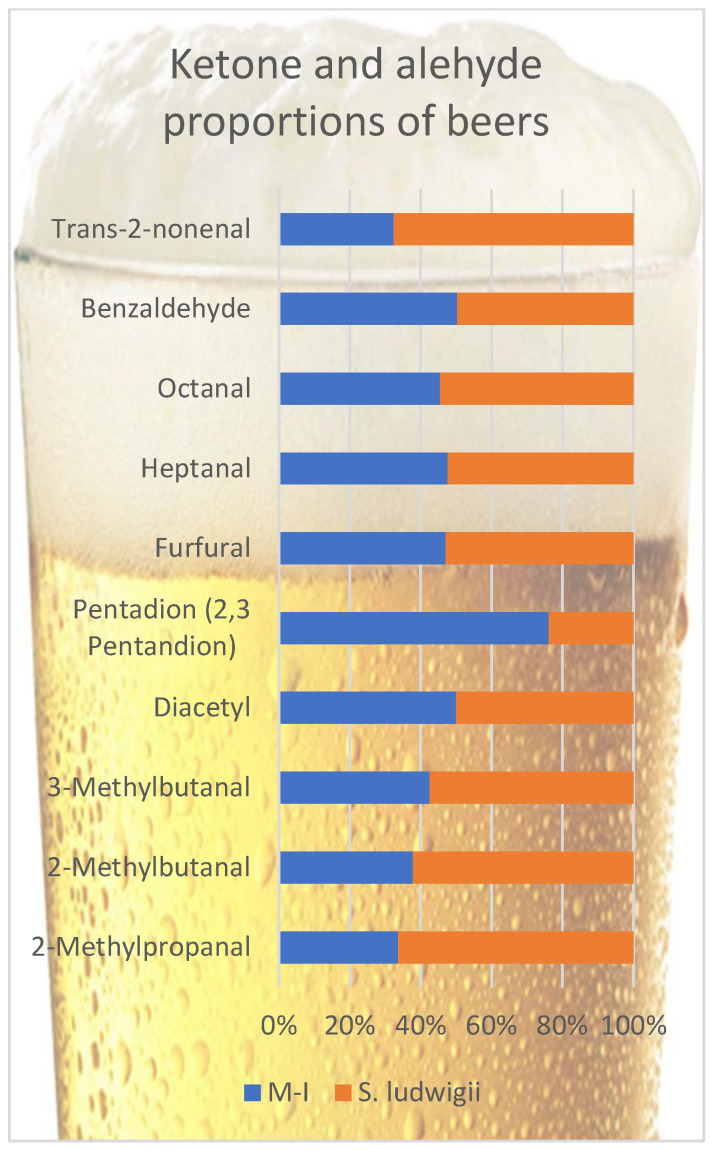
Comparison of some important off-flavors, aldehydes, and ketones in beer samples.

A part of the aldehydes form during the mashing and boiling, and with fermentation, additional aldehydes form as well [40]. The dissolution of aldehydes in ethanol causes a lower perception of wort taste, which is defined as “aldehyde retention” [53]. This aldehyde retention is about 32–39% in regular beers and only 8–12% in non-alcoholic beers [2]. In addition, brewer’s yeast may reduce the aldehyde content during re-fermentation to less aroma-active compounds, mainly corresponding alcohols [54]. A shorter fermentation time does not allow this aldehyde reduction, and the absence of ethanol and higher sugar concentration in wort (maltose) increases the worty flavor perception [11,53,55]. Figure 2 compares some important off-flavors, aldehydes, and ketones in beer samples.

The majority of these off-flavored compounds are aldehydes. All aldehyde compounds were detected at values higher than their perception thresholds. Among aldehydes, unpleasant–bitter–vinous-smelling compounds heptanal and octanal and “almond”-flavored compound benzaldehyde were found closer in both beer samples. Other aldehydes were higher in *S. ludwigii* than in the M-I beer sample. Among them, 2-methylpropanal, 2-methylbutanal, and 3-methylbutanal (Strecker aldehydes) can contribute to the ‘worty’ off-flavor [6,44,56,57]. An increase in the concentration of these Strecker aldehydes (also some esters) was related to flavor changes by aging [58]. Generally, even with different production methods, non-alcoholic beers are sensorially described as ‘worty’ [7,11]. Acetaldehyde is the upfront aldehyde in beer and is produced as an intermediate during ethanol production from carbohydrates, with green apple notes [59]. It has a 0.045 mg/L threshold value [6]. This study determined that the samples contained acetaldehyde, but the amount could not be determined.

According to these results, the flavor of M-I beer samples is expected to be less worty than *S. ludwigii* beer samples. (E)-2-nonenal, a degradation product of linoleic acid, is also described as a pivotal contributor to the stale flavor of beer, with its cardboard and papery notes [60]. One key factor is that M-I appears to minimize the formation and accumulation of these carbonyl compounds during fermentation. In conventional alcohol-free beer, these aldehydes accumulate from amino acid catabolism and are not adequately reduced, contributing to an undesirable wort-like aroma [6]. By contrast, the M-I strain likely has a higher aldehyde-reducing capacity—for example, it may more efficiently convert Strecker aldehydes into their corresponding alcohols (which are less flavor-active) using yeast dehydrogenases. In a recent study, a maltose-negative yeast strain exhibited a strong “yeast reducing power,” significantly lowering the levels of wort-derived aldehydes (e.g., methional and isovaleraldehyde) in the beer compared to *S. ludwigii* [61]. This superior reduction in aldehydes by M-I would explain the diminished 2-methylpropanal and 3-methylbutanal in its beers.

Vicinal diketones were lower in the M-I beer sample than in *S. ludwigii*. One is ‘buttery’ flavored; diacetyl (2,3-butanedione) has a low-threshold value off-flavor. Its amount may vary according to the fermentation conditions and the yeast type [2,62,63]. The other compound, 2,3-pentanedione, has a higher flavor threshold than diacetyl with a similar ‘butter’ but also a ‘toffee-like’ flavor [63]. M-I tends to produce less diacetyl, possibly because it excretes smaller amounts of α-acetolactate (the precursor of diacetyl) during amino acid synthesis and/or because any diacetyl that is formed is reabsorbed and metabolized more completely during maturation. Empirically, certain non-Saccharomyces brewing yeasts consistently keep diacetyl levels below sensory thresholds (≤0.1 mg/L) in finished low-alcohol beers [64]. The biochemical basis for M-I’s low diacetyl may lie in its limited maltose fermentation (restricting overproduction of valine-pathway intermediates) and potentially in enzymatic traits that expedite the reduction in vicinal diketones to flavorless end-products (acetoin/2,3-butanediol). Overall, these metabolic differences may contribute to a cleaner flavor profile in the M-I strain, potentially resulting in fewer aldehydic and vicinal diketone off-notes compared to *S. ludwigii* [6,61].

Volatile medium-chain fatty acids (hexanoic, octanoic, and decanoic acids) are considered important indicators for monitoring the maturation process in beer [25,65]. During fermentation and maturation, their levels can increase due to acid formation. They can impart a rancid, goat-like off-flavor if their concentrations rise significantly. The production of these acids can be influenced by wort composition, the yeast strain used, and fermentation conditions (such as aeration and temperature). Among these acids, hexanoic and octanoic acids were found at higher concentrations in the M-I sample compared to the *S. ludwigii* beer. In contrast, decanoic acid was found at a lower concentration. However, all volatile fatty acid levels were still much lower than their threshold values.

Overall, the observed differences in ester formation, higher alcohol production, and reduction of carbonyl compounds between M-I and *S. ludwigii* are likely linked to strain-specific metabolic pathways and enzymatic activities, which have been shown to strongly influence the volatile profile of non-alcoholic beers [18,61].

### 3.2. Sensory Analysis Results

The triangular test results are presented in Table 3. Among the 18 trained panelists, 10 correctly identified the different samples. The triangle test (*n* = 18) demonstrated that the assessors could distinguish the beers above chance (10/18 correct; exact binomial one-sided *p* = 0.043; ISO 4120:2004 - Methodology — Triangle test), indicating a statistically significant difference at the 0.05 level. These results confirm that the M-I and *S. ludwigii* samples were perceptibly different. In addition to the triangular test, a rating evaluation was conducted to assess each sample’s overall quality and preference. The panelists rated the M-I sample with an average score of 2.4, indicating a “very good beer” according to the 9-point scale used in the study. In contrast, the control sample (*S. ludwigii*) received an average score of 3.5, corresponding to “good beer.” While both samples were rated favorably, the M-I sample was perceived as slightly higher quality overall.

These results indicate that while some panelists detected the sensory differences between the two samples, the overall preference leaned towards the M-I sample.

**Figure 3:**
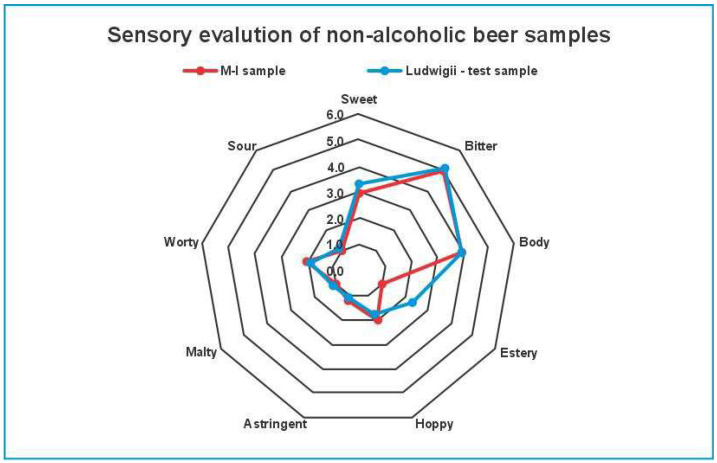
The sensorial profile test spider diagram of M-I (red) and control (*S. ludwigii*-blue) beer samples.

The sensorial profile test (Figure 3) revealed distinct differences between the M-I and control non-alcoholic beer samples (*S. ludwigii*) across several key sensory attributes. Panelists evaluated both samples based on sweetness, sourness, bitterness, body, estery aroma, hoppiness, maltiness, astringency, and worty characteristics. The sensorial profile test highlighted significant differences between the M-I and control samples (*S. ludwigii*), particularly in the attributes of esters and sweetness. The M-I sample was rated lower in estery aroma and sweetness, while the control sample (*S. ludwigii*) was perceived as more estery and slightly sweeter by the panelists. According to the Global Taste Score (GTS) results, the M-I beer sample received a score of 9.9, showing higher preference than the *S. ludwigii* beer samples, which scored 8.3. The interplay of compositional parameters can explain this situation: the slightly higher alcohol content, lower final extract, and overall ester levels detected in M-I (based on GC-MS data) likely contributed to a more balanced flavor perception. Moreover, the lower concentrations of carbonyl compounds (e.g., Strecker aldehydes) in the M-I sample may have reduced worty off-flavors, further supporting its higher acceptance. Even if panelists rated M-I as less estery in direct attribute scoring, its overall flavor integration—including reduced off-flavor notes—resulted in a more favorable impression.

Matrix effects and aroma interactions can explain the apparent discrepancy between chemical analysis and sensory perception of M-I beer. In complex beverages like beer, the chemical composition (the flavor matrix) can significantly alter how individual aroma compounds are perceived. This means a beer’s analytical profile (e.g., ester concentration) might not directly predict its sensory impact due to synergistic and masking interactions among volatiles [66]. For example, combinations of fruity esters can enhance overall fruit aroma beyond what each ester would produce alone, while certain other compounds can suppress or mask fruitiness [66].

Another factor to consider is residual sugar and sweetness. The slightly higher sweetness in the *S. ludwigii* beer may have further influenced aroma perception. Sweetness and fruit aromas can interact positively—a congruent sweet taste can make fruity aromas stand out more, and vice versa, fruity notes can enhance the impression of sweetness. Jackowski et al. [67] studied a beer fermented with a non-conventional yeast that had a higher perceived sweetness alongside its fruity flavor, attributed to the combination of residual sugar and ester-driven aroma [67]. In our context, the subtle sweetness of the *S. ludwigii* beer could be reinforcing its ester-derived fruitiness, making it seem “more estery” to the senses despite its lower total ester concentration. This interplay of taste and aroma underlines why a beer with a bit more residual sugar might present a stronger fruity/estery impression than a drier beer with more esters in chemistry alone.

## 4. Conclusions

The difference in alcohol content between M-I (0.42% vol.) and *S. ludwigii* (0.35% vol.) is likely due to variations in sugar metabolism and fermentation efficiency. M-I exhibited higher levels of esters (e.g., ethyl acetate, isoamyl acetate) and higher alcohols (e.g., 3-methyl-1-butanol, 2-phenylethanol), suggesting a more active fermentation process. Additionally, M-I’s lower apparent extract and density indicate greater sugar consumption.

The E/Ha ratio, representing the balance between total esters and higher alcohols, plays a crucial role in shaping beer’s overall sensory profile. A higher E/Ha ratio is typically associated with a more fruity and estery aroma profile, whereas lower ratios are linked to solvent-like, alcoholic notes that can negatively affect beer quality [50,68]. However, extremely high ratios may also result in an overpowering ester character and sensory imbalance. In the literature for *S. ludwigii* strain’s beer, ester/higher alcohol ratios (E/Ha mg/L) are generally found between 0.80/21.05 (E/Ha mg/L) [11] 14.91/43.31 (E/Ha mg/L) [15]. In this study, *the S. ludwigii strain*’s beer was generally higher than these ratios, with 3.88/7.62 mg/L. The M-I sample is higher than *S. ludwigii* strain’s beer with 8.41/15.12 mg/L. Generally, this rate of E/Ha in mg/L has been reported in a wide range for maltose-negative yeasts. For *Mrakia gelida (re-fermented DBVPG 5952)*, 3.52/27.7 mg/L is reported as having fruity apricot, grape, and litchi descriptors, but also with hop, cereal, malty, and caramel notes [69]; for *Pichia kudriavzevii*, 50/50 mg/L is reported as having relatively more desired volatiles in a balance [70]; for *Pichia kluvyeri* (PK-KR1), 25/20 mg/L reported that its flavor is very close to a beer containing alcohol of at least 4% (*v*/*v*) [71,72]. For *Cyberlindnera subsufficiens* (C6.1) 12.8/9.8 mg/L, which is described as pleasantly fruity with a bit of a worty-like character, it was found more pleasant than CBS 5763 strains with 7.82/21.03 E/Ha mg/L, which is described as fruity and pleasant [42]. In our study, both beers exhibited E/Ha ratios that fall within a moderate range, which can be associated with a fruity and pleasant flavor perception. This ratio is largely influenced by the yeast strain and fermentation conditions, highlighting the importance of strain selection and process optimization in modulating aroma-active compounds [50,68]. Recent studies have shown that in *Saccharomyces* and non-*Saccharomyces* species, the E/Ha ratio can be modulated not only by fermentation parameters (e.g., yeast strain, temperature, oxygen availability) but also through genetic regulation or modification of AATase genes (e.g., ATF1, ATF2), other genes involved in ester synthesis, and pathways related to amino acid catabolism (e.g., BAT2) [68].

According to volatile compound analysis, the M-I sample has a fruitier character than the *S. ludwigii* beer sample due to its higher amounts of esters and higher alcohol composition. Also, it has lower amounts of Strecker aldehydes, which can give a worty off-flavor. Although there were very slight differences in the taste parameters determined by the sensory analysis results, it was observed that the M-I beer was generally more preferred and showed great promise as a variety.

## Figures and Tables

**Table 1:** Physico-chemical parameters of non-alcoholic beers.

Physico-Chemical Parameters	M-I	Control—*S. ludwigii*
Original extract (% *w*/*w*)	5.34 ± 0.20	5.39 ± 0.20
Alcohol (% vol.)	0.42 ± 0.09	0.35 ± 0.09
Apparent extract (% *w*/*w*)	4.69 ± 0.30	4.84 ± 0.30
pH	4.76 ± 0.30	4.76 ± 0.30
Color (EBC)	7.85 ± 1.30	7.80 ± 1.30
Density (g/cm^3^)	1.01592	1.01679

Values expressed as mean ± standard deviation.

**Table 2:** The aroma analysis results of beers from M-I and *S. ludwigii*.

Substance	Concentration [mg/L]				
	M-I	*S. ludwigii*	Flavor in Beer	Perception Threshold (ppm)	References
**Acetate Esters**					
**Ethyl acetate**	6.86	3.41	Solvent, fruity, sweetish	21	[26]
**Propyl acetate**	0.01	0.002	Celery and raspberry	30	[27,28]
**Isobutyl acetate**	0.03	0.004	Fruit, solvent	1	[29]
**Isoamyl acetate**	0.66	0.06	Banana, apple, solvent, ester, pear	1.4	[26]
**2-Phenylethyl acetate**	0.31	0.05	Roses, honey, apple, sweetish	3.8	[30]
**Ethyl Esters**					
**Ethyl butyrate**	0.10	0.05	Papaya, cream, pineapple	0.4	[29]
**Ethyl hexanoate (caproate)**	0.27	0.20	Sour apple, aniseed	0.17	[31]
**Ethyl octanoate (caprylate)**	0.14	0.06	Sour Apple	0.3	[31]
**Ethyl decanoate (caprate)**	0.03	0.04	Brandy	1.5	[27,28]
**Higher Alcohols**					
**2-Methyl-1-propanol**	1.24	0.81	Alcohol	200	[32]
**3-Methyl-1-butanol**	4.51	1.75	Alcohol	70	[32]
**2-Methyl-1-butanol**	2.32	1.42	Alcohol, banana, medicinal, solvent	65	[25]
**2-Phenylethanol**	7.05	3.64	Roses, sweetish, perfumed	125	[32]
**Hop Derived Terpenoid**					
**Linalool**	0.03	0.03	Citrus, floral	0.08	[27,33]
**Aldehydes**					
**2-Methylpropanal**	11.56	22.75	Grainy, varnish, fruity	1	[34,35]
**2-Methylbutanal**	4.62	7.59	Almond, apple-like, malty	1.25	[27,34]
**3-Methylbutanal**	13.63	18.40	Malty, cherry, almond, chocolate	0.6	[34,35]
**Heptanal**	0.30	0.33	Fat, citrus, rancid	0.08	[27,36]
**Octanal**	0.15	0.18	Fat, soap, lemon, green	0.04	[27,36]
**Benzaldehyde**	8.83	8.70	Almond-like	2	[27,37]
**Trans-2-nonenal**	0.12	0.25	Cardboard, papery, cucumber	0.00011	[27,38]
**Vicinal Diketones**					
**Diacetyl**	33.34	33.42	Butterscotch	0.15	[32]
**Pentadion (2,3 Pentandion)**	34.23	10.74	Butterscotch and toffee	0.9	[27,37]
**Volatile Fatty Acids**					
**Hexanoic acid (caproic)**	1.09	0.18	Goaty, fatty acid	8	[25]
**Octanoic acid (caprylic)**	1.36	0.30	Goaty, fatty acid	14	[39]
**Decanoic acid (capric)**	0.29	0.40	Waxy, rancid	10	[39]

**Table 3:** The triangular test and rating results of non-alcoholic beers from M-I and *S. ludwigii*.

Number of Tasters	Correct Answers ^a^	Result	M-I Sample Rating ^b^	*S. ludwigii* Sample Rating ^b^
18	10 from 18	There is a sensory difference between the samples	2.4	3.5

^a^ Conclusiveness = % of correct answers from total answers. ^b^ Rating: 1—excellent beer, 2– very good beer, 3—good beer, 4—relatively good beer, 5—acceptable beer, 6—relatively bad beer, 7—bad beer, 8—very bad beer, 9—horrible beer.

## Data Availability

The original contributions presented in the study are included in the article, further inquiries can be directed to the corresponding author.
